# Exploring the mechanism by which *Angelica sinensis* improves haematopoietic function in aplastic anaemia

**DOI:** 10.18632/aging.205971

**Published:** 2024-06-26

**Authors:** Zetao Chen, Li Cheng, Jing Zhang, Xing Cui

**Affiliations:** 1 Department of Gerontology, Affiliated Hospital of Shandong University of Traditional Chinese Medicine, Jinan 250014, China; 2 Department of Acupuncture, Affiliated Hospital of Shandong University of Traditional Chinese Medicine, Jinan 250014, China; 3 Department of Science and Education, Shandong Mental Health Center, Jinan 250014, China; 4 Department of Hematology, Affiliated Hospital of Shandong University of Traditional Chinese Medicine, Jinan 250014, China

**Keywords:** aplastic anaemia, apoptosis, *Angelica sinensis*, *Angelica sinensis* polysaccharide, Th17 cell, IL-17A, Bax

## Abstract

*Angelica sinensis* (AS) can improve the haematopoietic function, but the treatment mechanism is unknown. Transfusion dependency was estimated by Kaplan–Meier survival analyses and Cox proportional-hazard model in AS treated apalstic anemia (AA) patients. After that, the AA GEO database was analysed, the up differentially expressed genes (DEGs) of AA were combined with AS targets for the intersection of targets. After the AA mouse model was established, the effect of AS was confirmed by haematopoietic function tests. The same experiment plus mitochondrial apoptotic pathway tests *in vivo* were performed in *Angelica sinensis* polysaccharide (ASP)-treated mice, the key ingredient in AS. For *in vitro* experiment, bone marrow nucleated cells (BMNCs) were tested. Clinical data confirmed that the level of transfusion dependency and IL17A were lower in AS-users compared to non-AS users (*p* < 0.001). The intersection of targets between AA and AS most concentrated on inflammation and apoptosis. Then, the same effect was found in AS treated AA mice model. In both *in vivo* and *in vitro* tests, ASP demonstrated the ability to mitigate P38/MAPK-induced Bax-associated mitochondrial apoptosis, while also reducing the levels of activated Th17 cells and alleviating abnormal cytokine levels. So, the protective effect of AS and ASP on hematopoietic function lies in their ability to prevent apoptosis.

## INTRODUCTION

Immunosuppressive therapy (IST) is the primary treatment for patients diagnosed with aplastic anemia (AA). Despite the common use of Cyclosporin A (CsA) in IST, which effectively regulates T cell-mediated autoimmune response by modulating Ca2+ or calmodulin-dependent phosphatase activity and inhibiting IFN, TNF, and IL-2 production from T cells, thereby suppressing the negative regulation of hematopoietic function caused by these lymphoid factors [1], a significant proportion of AA patients still exhibits resistance to IST (30%). Multiple studies have confirmed that the excessive activation of IL-17-producing (Th17) CD4+ T cells plays a crucial role by promoting increased inflammatory response [2, 3] and damaging haematopoietic function [4] in the pathogenesis of AA.

*Angelica sinensis* (AS) is used extensively in traditional Chinese medicine (TCM), which could enhance the production of blood cells and promote the proliferation of bone marrow nucleated cells (BMNCs) [5]. In patients with aplastic anemia, AS can reduce the risks of overall and anemia-related mortalities [6]. AS and some of its main active ingredients, such as *Angelica organic* acid [7], glabralactone [8] and *Angelica sinensis* polysaccharide (ASP) can reduce inflammation and reverses anemia [9, 10], ASP can also improve the haematopoietic function of CD34+ haematopoietic stem/progenitor cells (HSPCs) [11]. The administration of ASP has been shown to enhance the protection of haematopoietic stem cells in an X-ray irradiation-induced ageing model. This is achieved through the upregulation of telomere length [12], as well as the expression of sirt1 and FoxO1 [13]. Additionally, ASP treatment leads to an increase in thymus and spleen index [14] while reducing oxidative stress levels [13].

The aim of this study was to investigate the impact of AS on AA patients and elucidate the mechanism underlying its effects and active constituents.

## METHODS

### Materials

The Radix *Angelica sinensis* (origin, Gansu) was purchased from the Pharmacy of Shandong University of Traditional Chinese Medicine Affiliated Hospital (Jinan, China) and identified by the pharmacist. ASP with >98% purity was purchased from Ci Yuan Biotechnology Co., Ltd., Shanxi (Xian, China). PrestoBlue^™^ Cell Viability Reagent was purchased from Thermo Fisher Scientific (Shanghai, China). Reactive oxygen species (ROS) assay kit was obtained from Wuhan Boster Biotechnology, Ltd. (Wuhan, China). The Annexin V-FITC/PI kit was purchased from Nanjing Jiancheng Bioengineering Institute (Nanjing, China). Cleaved caspase-9, caspase-9, cleaved caspase-3, caspase-3, Bad, Bax, Bcl-2, AIF, Cyt-c, p-p38, and p38 were purchased from Cell Signalling Technology (USA). TGF beta 1 antibody was purchased from Abcam Co. (Cambridge, UK). IL-17a polyclonal antibody was purchased from Proteintech (Wuhan, China). FITC-labelled antibodies against lineage markers were purchased from BD Biosciences (Shanghai, China).

### Clinical serum specimens

83 patients with chronic AA, who were treated in the Department of Hematology, Shandong University of Traditional Chinese Medicine Affiliated Hospital (Jinan, China) from August 2016 to February 2018, were used to investigate the frequency of transfusion dependency between AS and non-AS treatment. Patients who were treated by traditional Chinese medicine formula involving AS with standard medicine like cyclosporin A and/or stanozolol were named as AS group, otherwise, those patients who were treated with cyclosporin A and/or stanozolol were divided into control group. The peripheral blood samples were collected in evacuated and coagulation-promoting tubes, and serum samples were obtained after centrifugation (5 minutes, 2500 rpm). The primers of IL17 were 5′-GTTAGGGTGCTTTAGGTCC-3′ (forward), 5′-TAACAATGAGTTTCTGTACG-3′ (reverse). The study was approved by the ethics committee of the Shandong University of Traditional Chinese Medicine Affiliated Hospital, and all participants signed informed consent forms before the test.

### DEGs screening of AA

The row gene expression data between the normal group and AA group (GSE3807) were obtained from the Gene Expression Omnibus dataset (GEO, https://www.ncbi.nlm.nih.gov/geo/). The differentially expressed genes (DEGs) between AA and healthy donors were found using the R packages “limma” and “heatmap”, genes with an adjusted *P* < 0.05 and |logFold Change (FC)| >1 were considered DEGs and AA-related targets. To further reveal the potential function of DEGs, gene set enrichment analysis (GSEA) was performed. According to |logFC| >1, the rank list for the GSEA was created.

### Network pharmacology analysis of AS

The core active component structures of AS were obtained from PubChem (https://pubchem.ncbi.nlm.nih.gov/) platforms. Then, 6 databases, including ChEMBL (https://www.ebi.ac.uk/chembl/), CTD (https://ctdbase.org), TCMSP (https://old.tcmsp-e.com/tcmsp.php), ETCM (http://www.tcmip.cn/ETCM/index.php), BATMAN (http://bionet.ncpsb.org.cn/batman-tcm/), and SwissTargetPrediction (http://old.swisstargetprediction.ch/), were used to search for the potential targets of AS. After that, the intersection of targets from AA and AS was visualized using the Venny online tool (https://bioinformatics.psb.ugent.be/webtools/Venn/). The protein–protein interaction (PPI) network of the therapeutic targets between AA up DEGs and AS was constructed using the STRING online platform (https://string-db.org/), and the visualization was presented using Cytoscape 3.8.2.

### AA mice model and drug treatment

BALB/c mice (male, 6–8 weeks, 18–22 g) were purchased in the Experimental Animal Center of Shandong University (China). The mice were randomly divided into groups as follows: the normal control group, the model group, three different dose AS treated groups and three different dose ASP treated groups. The AA model was established as previously described [15], the mice were irradiated with 5.0 Gy Co60 γ-radiation, and then 2 × 10^6^ lymph node cells from DBA/2 donor mice were transplanted within 4 h of irradiation.

For the AS treated groups, mice were intragastrically fed with AS (AS is commonly administered at 9–15 g per day for human. Therefore, the medium dose we administered was 10 g per day. The converted dose for mice should be: 0.65 g/kg/day, 1.3 g/kg/day, and 2.6 g/kg/day). For the ASP treated groups we intragastrically fed ASP (100, 200, and 400 mg/kg/d), according to Mu’s study [14] and our preliminary experiment. The mice in the normal control and model groups were intragastrically fed a diet supplemented with physiological saline (10 ml/kg/d). After treatment for 2 weeks, euthanasia was performed using cervical dislocation on day 14, blood, femur and spleen were collected.

### Analysis of T-cell subsets and cytokines

The spleen is aseptically placed on a cell culture plate and mechanically disrupted using a cell filter to generate a single-cell suspension, typically with the addition of erythrocyte lysate buffer for efficient removal of red blood cells. Then cells were incubated with antibodies on ice for 30 min, the CD4+CD25-CD62L+CD44- population, which consisted of the initial CD4+T cells, was isolated. The corresponding cytokines were added to the medium to facilitate *in vitro* differentiation of Th1/Th2/Treg/Th17 cells. Stimulation with 10 ng/ml PMA and 1000 ng/ml Ionomycin was performed for 4 hours. Subsequently, flow cytometry was used to label specific intracellular cytokine markers for each subgroup and determine their composition.

The serum levels of interleukin-17A (IL-17A) and transforming growth factor-β (TGF-β) were detected using ELISA kits (Hengyuan, Shanghai, China). The OD value was measured at 450 nm using a microplate reader (Thermo Fisher Scientific, Rockford, IL, USA).

### ROS staining

BMNC cells were collected at the selected time point. One milliliter of the fluorescent probe DCFH-DA was diluted to 1:1000 added to the cells, which were then incubated at 37°C for 30 min. The cells were then washed with PBS buffer, counterstained with DAPI, and washed. A confocal laser scanning microscope was used to image the cells and determine the distribution and expression of ROS.

### Analysis of apoptosis

Using flow cytometry, we measured the apoptosis of BMNC cells. For flow cytometry, cardiomyocytes were collected and incubated for 20 min with Annexin V-FITC and 7-AAD. After that, the apoptosis rates were determined by flow cytometry.

### Immunohistochemistry staining analysis of bone marrow biopsy

Bone marrow biopsy tissues were fixed with 70% ethanol overnight and then embedded in paraffin. After incubating with primary antibody in a moisture chamber at 4°C overnight, the sections were incubated with secondary antibody for 60 min at 37°C. Then, they were incubated with avidin-biotinylated peroxidase complex, and peroxidase activity was exposed using DAB.

### *In vitro* experiments

For the *in vitro* experiments, BMNCs were obtained from mice at d2. After that, samples from the control and model group were cultured in IMDM with 10% FBS. The treated group was cultured in the same medium containing ASP (100, 200 and 400 ng/ml).

### Western blot analysis

Total protein was collected after lysis of the BMNCs. The protein was subjected to 10% sodium dodecyl sulfate polyacrylamide gel electrophoresis (SDS-PAGE). The proteins were then transferred electrophoretically to 0.45-μm nitrocellulose membranes, and the membranes were incubated overnight with primary antibodies at 4°C and then with secondary antibodies. The band intensities were quantified using ImageJ Software.

### Mitochondrial analyses by TEM morphometry

Mitochondrial structure was analyzed by electron microscopy. Then, images were obtained using a digital video camera. The quantitative density parameters (volume density (Vv) and numerical density (Nv)) and average surface area (S) were calculated.

### Colocalization analysis of mitochondria with Bax *in vitro*

The colocalization of mitochondria with Bax was observed by confocal fluorescence microscopy. For labeling the mitochondria, MitoTracker^™^ Red CMXRos (Thermo Fisher Scientific, M7512) (500 nM) was added to living cells. The cell slides were incubated with Bax antibodies (1:100) at 4°C overnight. After incubation with secondary antibody, the slides were analyzed by fluorescence microscopy at 550 nm for MitoTracker^™^ Red CMXRos and the respective wavelengths for the fluorescence secondary antibodies.

### Statistical analysis

The experimental results were statistically analyzed using SPSS 19.0 and GraphPad Prism 8.0 (Dotmatics, Boston, MA, USA). If each group of data had a normal distribution and homogeneity of variance, quantitative data were presented as the mean ± standard deviation (x̄ ± s). Differences between two groups were analyzed by Student’s *t*-test, and differences among multiple groups were analyzed by one-way ANOVA. Statistically significant changes were classified as significant if *P*-value is less than 0.05.

### Availability of data and material

The datasets supporting the conclusions of this article are included within the article. The datasets used and/or analyzed during the current study are available from the corresponding author on reasonable request.

## RESULTS

### Demographic characteristics and transfusion dependency analysis

**Figure 1:**
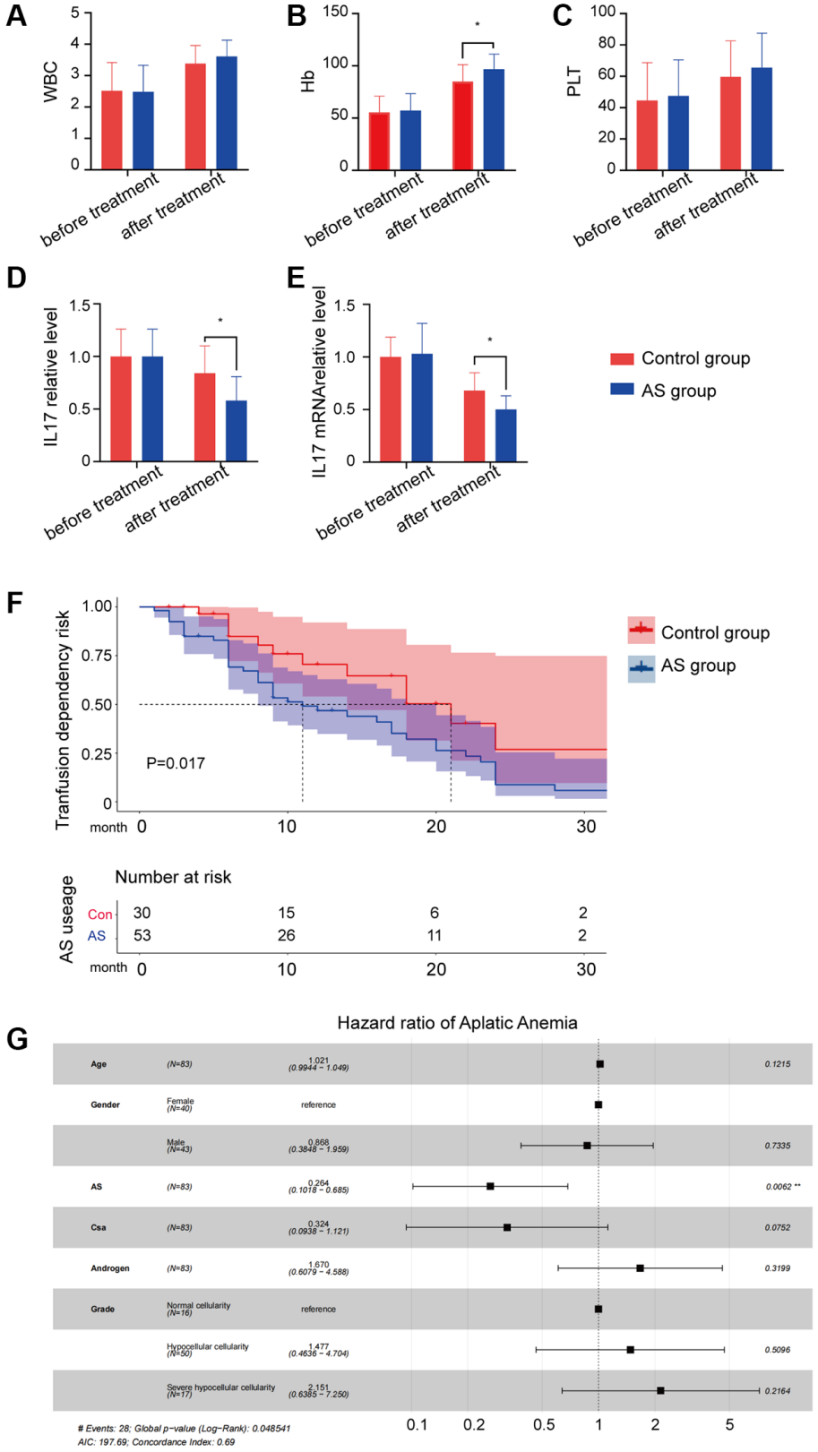
**The result of AS treatment.** (**A**–**C**) Peripheral blood cell counts in different groups. (**D**) IL17A protein level in different groups. (**E**) IL17A mRNA level in different groups. (**F**) Transfusion dependency risk in different groups. (**G**) Hazard ratio of different characters in aplastic anemia patients.

53 AS users (AS group) and 30 non-AS users (control group) were enrolled in this study. Blood transfusion therapy was significantly different between AS and non-AS users (*p* < 0.05; Table 1). After confirmed that AS can increase peripheral blood cells especially hemoglobin (Figure 1A–1C) and alleviate the level of IL17 protein and mRNA (Figure 1D, 1E), to evaluate the effect of the efficacy of AS on transfusion dependency, the crude and adjusted Cox proportional hazard models were performed in patients with AA (Figure 1F). We observed that patients with AS use had the statistical significance of a lower level of transfusion dependency (aHR: 0.70, 95% CI: 0.66–0.74, *p* < 0.001; Figure 1A) and a lower level of IL17A (Figure 1G).

**Table 1:** Characteristics of clinical data.

	**Level**	**Overall**	**No**	**Yes**	***p***
*n*		83	30	53	
Sex (%)	Female	40 (48.19)	13 (43.33)	27 (50.94)	0.6614
	Male	43 (51.81)	17 (56.67)	26 (49.06)	
Age (%)	>0, <18	2 (2.41)	0 (0.00)	2 (3.77)	0.4821
	≥18, <65	24 (28.92)	10 (33.33)	14 (26.42)	
	≥18, <40	51 (61.45)	19 (63.33)	32 (60.38)	
	≥65	6 (7.23)	1 (3.33)	5 (9.43)	
AS (%)	No	30 (36.14)	30 (100.00)	0 (0.00)	<0.0001
	Yes	53 (63.86)	0 (0.00)	53 (100.00)	
CsA (%)	No	18 (21.69)	0 (0.00)	18 (33.96)	0.0009
	Yes	65 (78.31)	30 (100.00)	35 (66.04)	
Androgen (%)	No	26 (31.33)	8 (26.67)	18 (33.96)	0.6584
	Yes	57 (68.67)	22 (73.33)	35 (66.04)	
Blood Transfusion Dependency (%)	No	55 (66.27)	13 (43.33)	42 (79.25)	0.0021
	Yes	28 (33.73)	17 (56.67)	11 (20.75)	
The grade of cellularity in bone marrow (%)	Normal	16 (19.28)	5 (16.67)	11 (20.75)	0.8843
	Hypocellular	50 (60.24)	19 (63.33)	31 (58.49)	
	Severe hypocellular	17 (20.48)	6 (20.00)	11 (20.75)	

### GO and GSEA analyses of DEGs of the AA cohort

**Figure 2:**
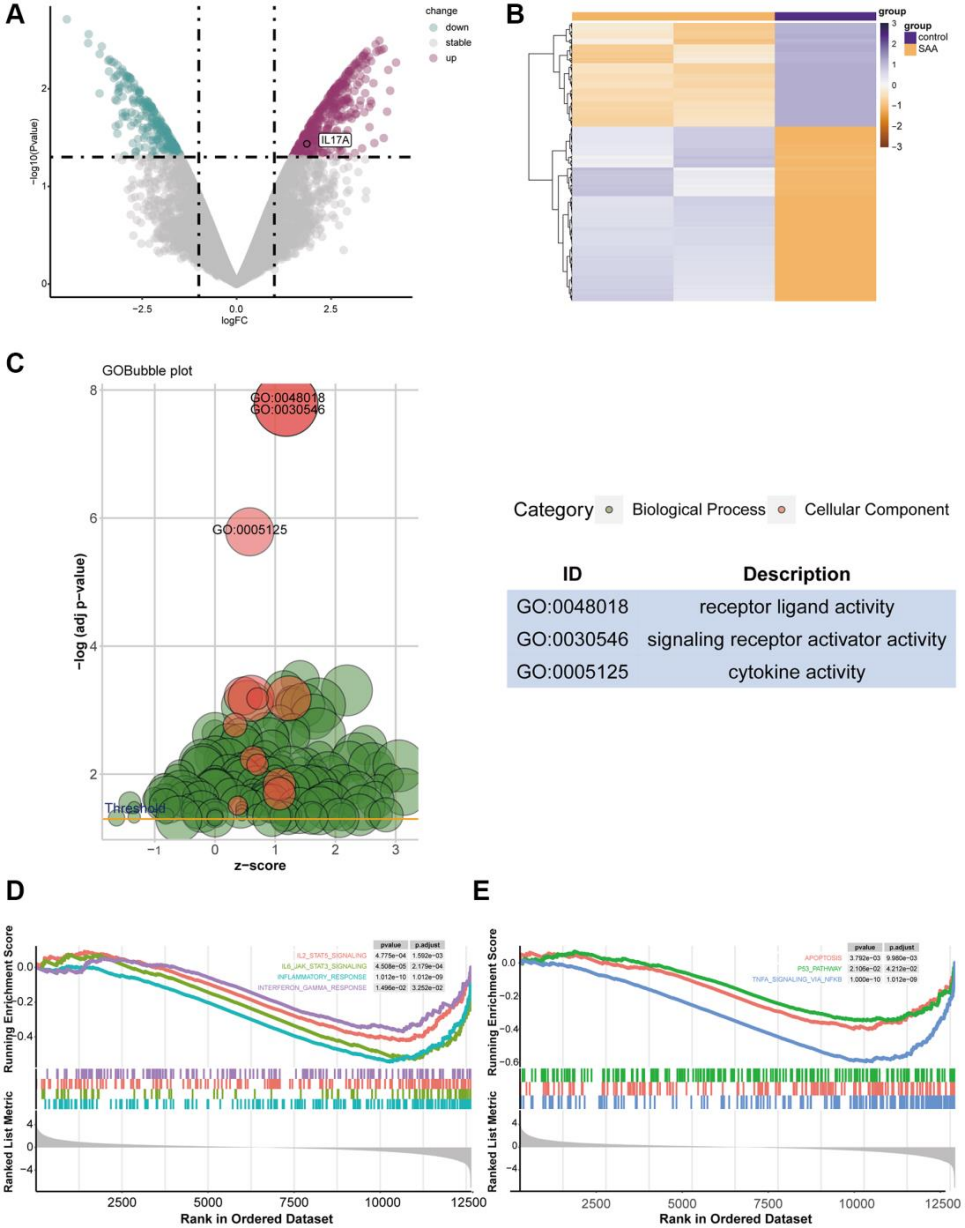
**The result of DEG identification.** (**A**) DEGs in GSE3807. (**B**) Heatmap of 1089 DEGs screened. (**C**) Gene Ontology (GO) enrichment analysis for DEGs. (**D**, **E**) Gene set enrichment analysis (GSEA) of DEGs.

The GEO database provided the raw data for GSE3807, and Table 2 displays the general features of the samples. A total of 1089 significant DEGs related to AA (*p*-value < 0.05, |logFC| >1.5) were found, with upregulated IL-17A and TGFBR1 (Figure 2A). The DEGs distribution heatmap showed evidently different expression between the two groups (Figure 2B). After 775 GO terms were obtained (Figure 2C), GSEA enrichment analysis showed that immune-related and apoptosis-related pathways, such as IL2-STAT5, IL6-JAK-STAT3, inflammatory response, IFN-γ, TNF-α, apoptosis and P53, were activated (Figure 2D, 2E).

**Table 2:** Characteristics of GEO data samples.

**Sample**	**Age/Sex**	**Categories of samples**	**Tissue/Cell type**
1	19/F	Aplastic anemia	bone marrow/CD3+ T cells
2	40/F	Aplastic anemia	bone marrow/CD3+ T cells
3	57/F	Aplastic anemia	bone marrow/CD3+ T cells
4	64/M	Aplastic anemia	bone marrow/CD3+ T cells
5	70/F	Aplastic anemia	bone marrow/CD3+ T cells
6	70/M	Aplastic anemia	bone marrow/CD3+ T cells
7	42/F	Healthy control	bone marrow/CD3+ T cells
8	58/M	Healthy control	bone marrow/CD3+ T cells

### AS-AA target network and GO, KEGG enrichment analysis

**Figure 3:**
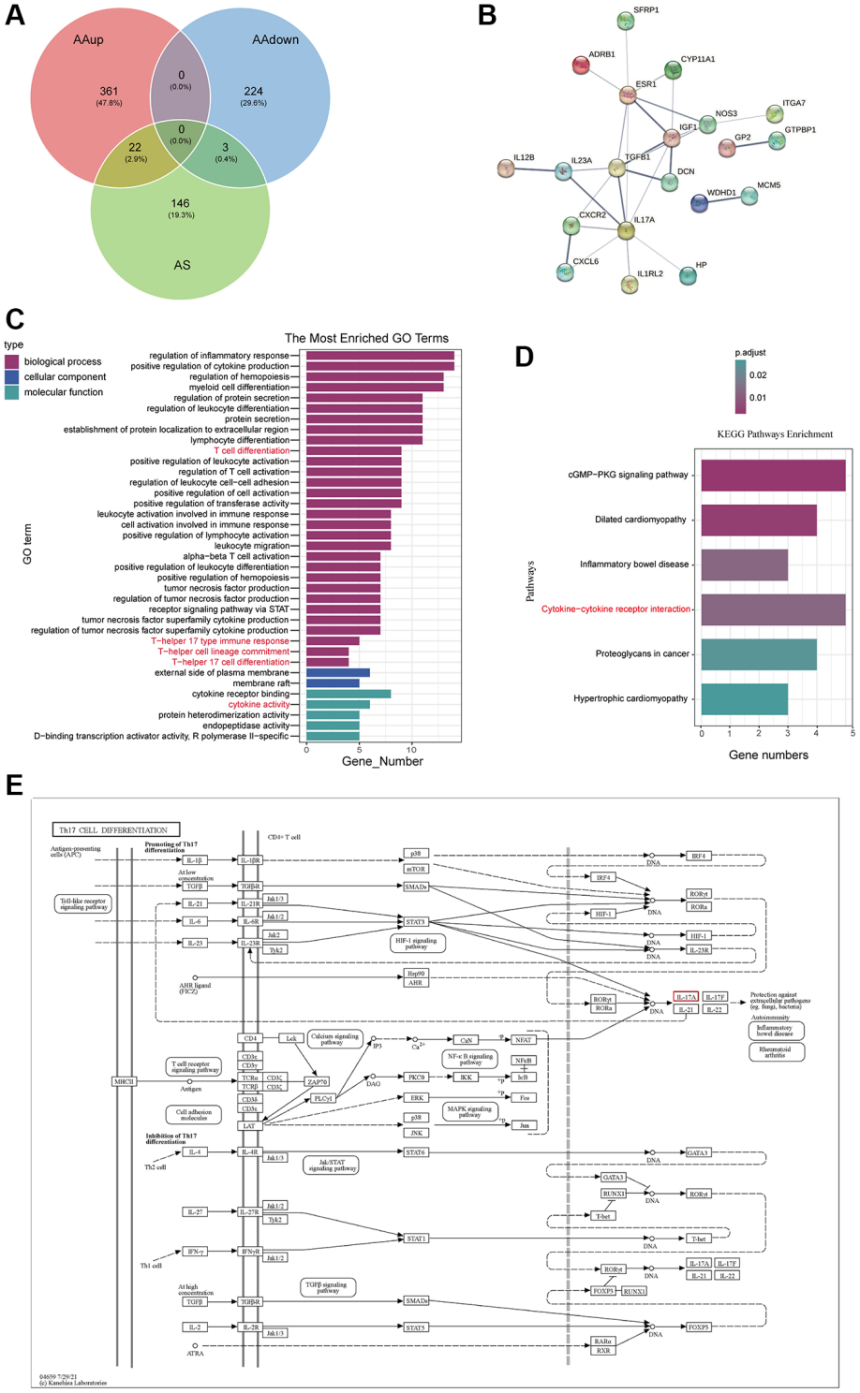
**AS-AA target network and GO, KEGG enrichment analysis.** (**A**) Venn diagram of the targets in AA and AS. (**B**) PPI network. (**C**) GO function enrichment analysis. (**D**) KEGG pathway enrichment analysis of potential therapeutic targets. (**E**) Network of KEGG pathways and potential therapeutic targets.

25 potential targets for AS treatment of AA were identified with a Venn diagram (Figure 3A). Next, STRING Plapliform was used to analyse protein–protein interactions, and Cytoscape was used to visualize predicted targets of AS in the treatment of AA (Figure 3B). Our GO and KEGG analysis of up-regulated genes in AA that could be targeted by AS revealed that the enriched GO terms mainly involved the regulation of inflammation response, cytokine activity, and T cell differentiation, particularly T-helper 17 cell differentiation and T-helper 17 type immune response (Figure 3C). The KEGG pathway enrichment analysis identified potential therapeutic pathways, including the cytokine-cytokine receptor interaction pathway (Figure 3D). These findings suggest that AS primarily treats AA by modulating aberrant immunotherapy. In support of this, we presented a signaling pathway diagram of Th17 cell differentiation and its related cytokines (Figure 3E).

### Effects of AS treatment on enhancing haematopoietic function and regulating the abnormal Treg/Th17 ratio in AA mice

**Figure 4:**
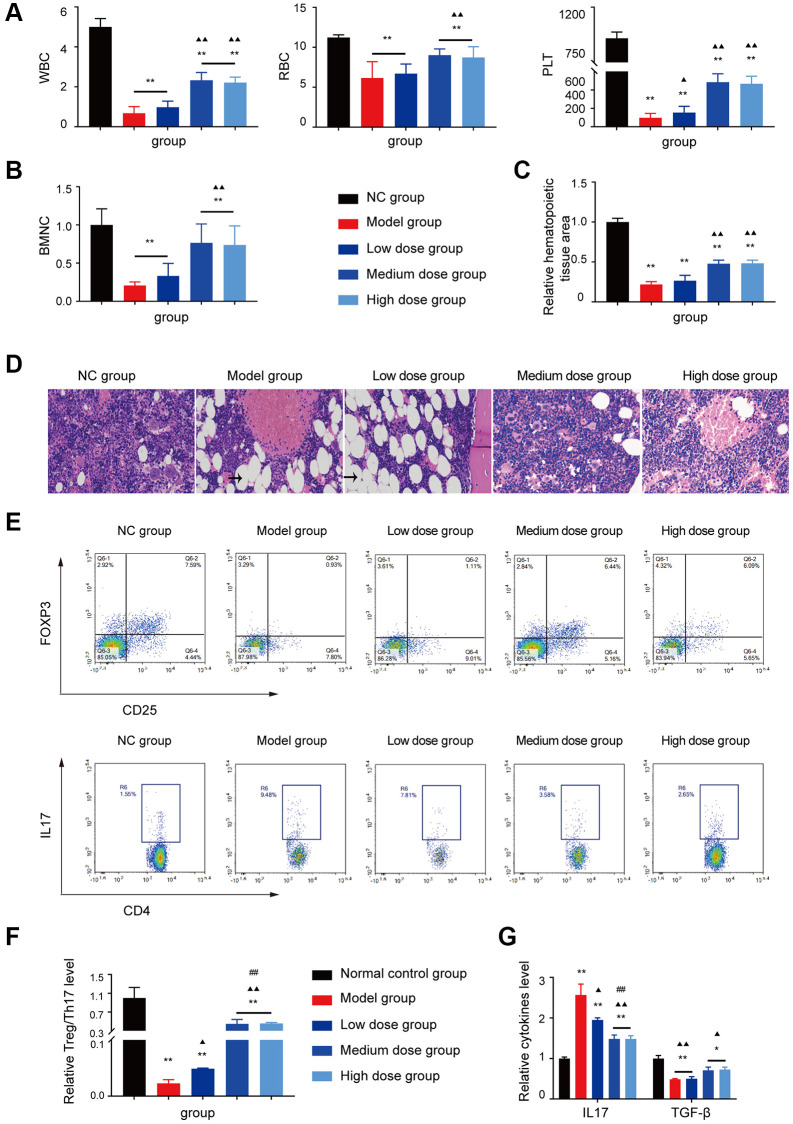
**Analysis of AS in treatment of AA.** (**A**, **B**) Peripheral blood cell and BMNCs counts in the different groups. (**C**, **D**) Hematopoietic tissue assay and bone marrow biopsy of different mice groups on day 14 (×400). (**E**) Treg and Th17 cells were detected using FCM. (**F**) The Treg/Th17 ratio in different AA groups. (**G**) The levels of IL-17A and TGF-β in different groups. Note: ^*^P < 0.05 and ^**^P < 0.01 compared with the normal control group. ^▲^*P* < 0.05 and ^▲▲^*P* < 0.01 compared with the model control group, ^#^*P* < 0.05 and ^##^*P* < 0.01 compared with the low-dose group.

The WBC/RBC/PLT level, BMNCs counts and the relative haematopoietic tissue area levels of the medium dose and high dose treated groups were increased compared with the model group (*P* < 0.05 or *P* < 0.01; Figure 4A–4D).

The hyperinflammatory environment in severe AA is triggered by increased levels of IL-17 and IL-1β, along with decreased levels of TGF-β [16, 17]. Furthermore, the single-cell profiling revealed the population of Th17-polarized CD4+CAMK4+ naïve T cells that exhibit activation of the IL-6/JAK3/STAT3 pathway, indicating a predisposition to proinflammatory pathogenesis in severe AA [18]. Based on the analysis of GEO data and the abnormal T cell subset research in AA, the Treg/Th17 ratio, IL17 level, and TGF-β level were tested, after AS treatment, the Th17 cell related aberrant immune function was corrected by AS as shown in Figure 4E–4G, which indicated that AS could improve mice haematopoietic fuction like in AA patients.

### Effects of AS on ROS and mitochondrial apoptosis pathway

**Figure 5:**
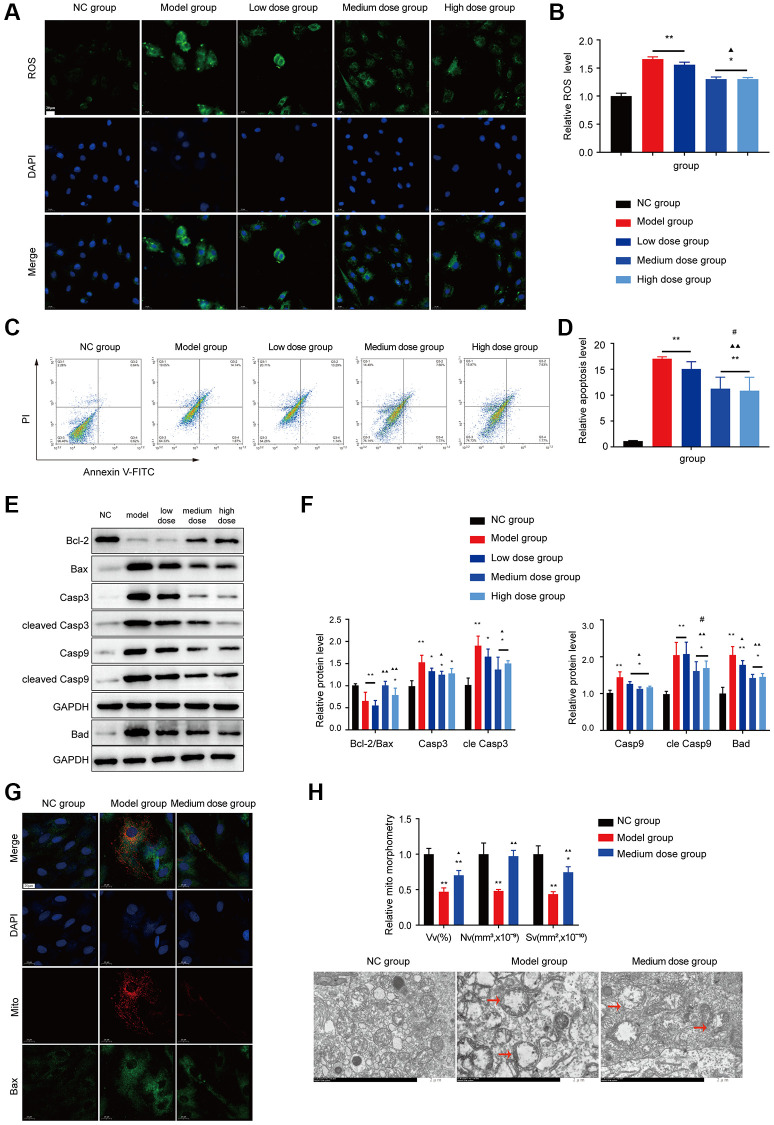
**Effects of AS on apoptosis in BMMC cells.** (**A**) Immunofluorescence images of ROS. (**B**) The ROS levels of each group were assessed. (**C**) Flow cytometry scatter plot of apoptosis. (**D**) Apoptosis was assessed in different groups. (**E**, **F**) Markers of apoptosis were tested using Western blotting. (**G**) The colocalization of Bax with mitochondria (yellow dots). (**H**) Mitochondrial structure was detected by electron microscopy. Note: ^*^P < 0.05 and ^**^P < 0.01 compared with the normal control group. ^▲^*P* < 0.05 and ^▲▲^*P* < 0.01 compared with the model control group, ^#^*P* < 0.05 and ^##^*P* < 0.01 compared with the low-dose group.

The results of ROS showed that medium dose AS significantly decreased intracellular ROS levels compared with the model control group (Figure 5A, 5B).

As shown in Figure 5C, 5D, AS treatment decreased the BMNCs apoptosis ratio compared with that of the model group (*P* < 0.01), and WB also verified the AS can alleviate the apoptosis level (Figure 5E, 5F). AS also could reduce the Bax mitochondrial translocation (Figure 5G). Transmission electron microscopy showed that mitochondria in the model showed crista breakage or disappearance (Figure 5H), after treatment with AS, the structure of mitochondria was improved, suggesting that AS could block apoptosis and improve mitochondrial damage.

### Effects of ASP in AA mice

**Figure 6:**
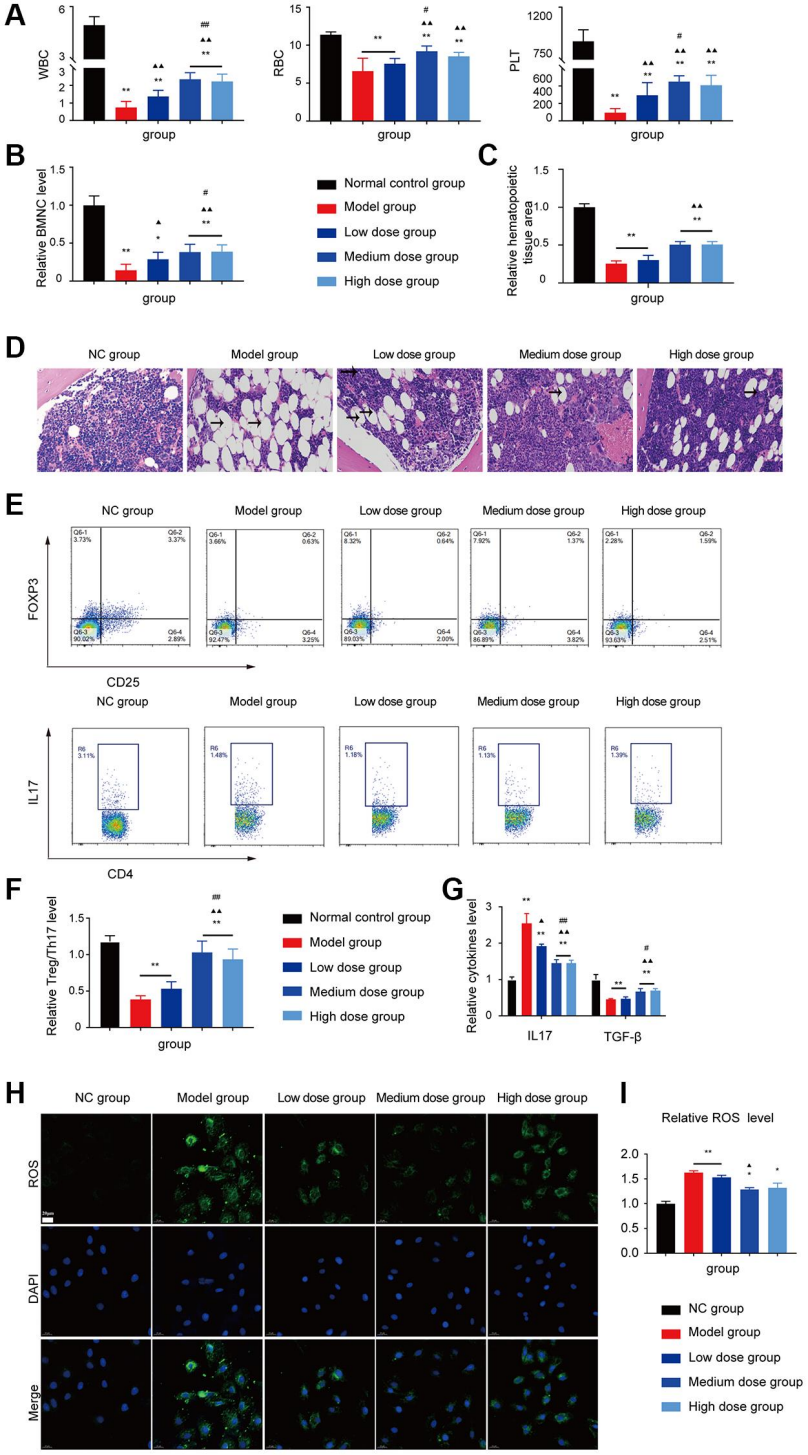
**Assessment of the ASP treatment in the AA mice.** (**A**) Peripheral blood cell counts in the different groups. (**B**) BMNC counts assay in the different groups. (**C**, **D**) Hematopoietic tissue assay and bone marrow biopsy of different mice groups on day 14 (×400). (**E**) Treg and Th17 cells were detected using FCM. (**F**) The Treg/Th17 ratio in different AA groups. (**G**) The levels of IL-17A and TGF-β in different groups. (**H**) Immunofluorescence images of ROS. (**I**) The ROS levels of each group were assessed. Note: ^*^P < 0.05 and ^**^P < 0.01 compared with the normal control group. ^▲^*P* < 0.05 and ^▲▲^*P* < 0.01 compared with the model control group, ^#^*P* < 0.05 and ^##^*P* < 0.01 compared with the low-dose group.

The WBC/RBC/PLT level, BMNC counts and the relative haematopoietic tissue area levels of the medium dose and high dose treated groups were increased compared with the model group (*P* < 0.05 or *P* < 0.01; Figure 6A–6D). As shown in Figure 6E–6I, the Treg/Th17 ratio, IL17 level, TGF-β level and the ROS level in the AA mice recovered after ASP treatment.

**Figure 7:**
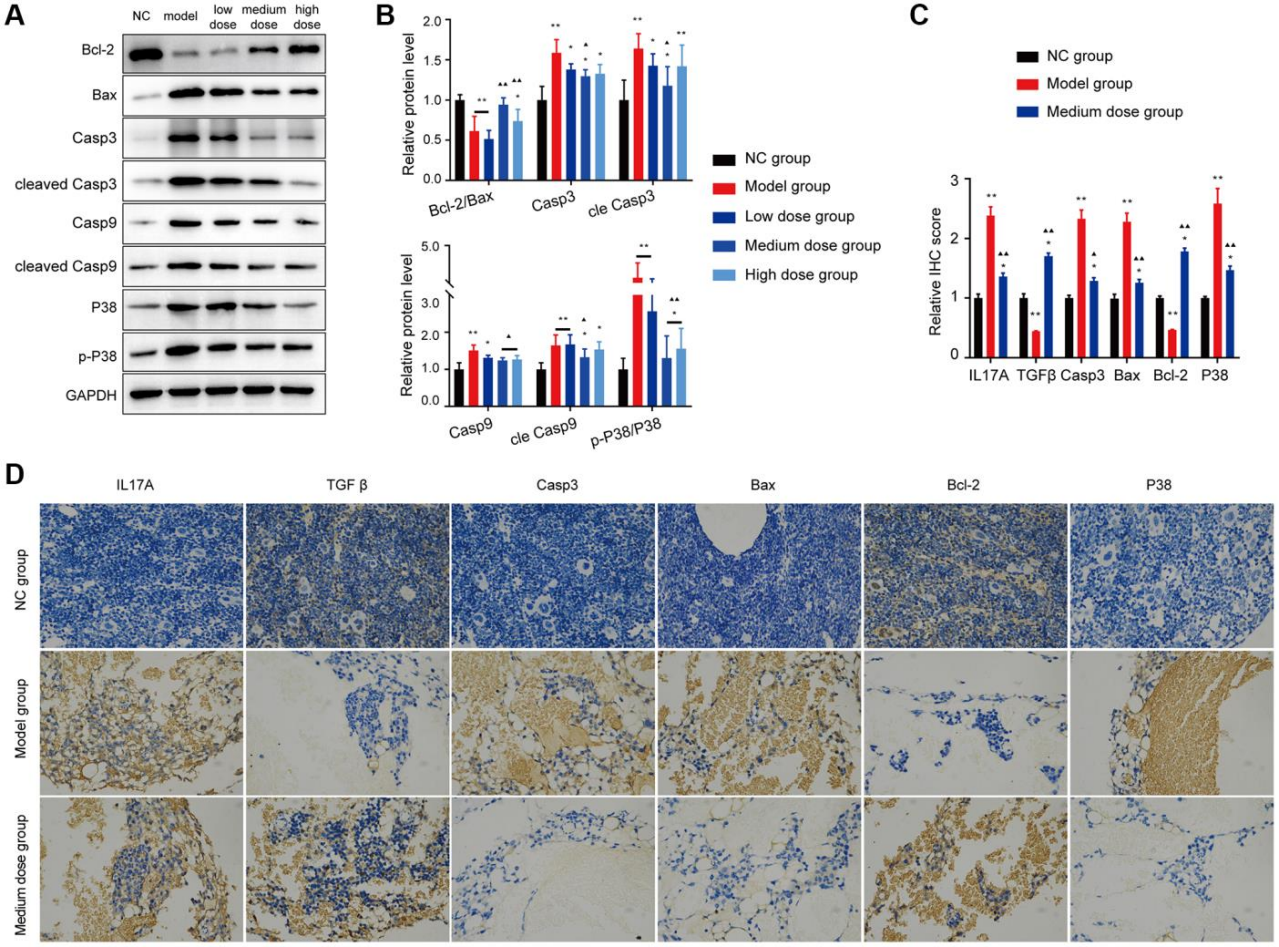
**Effects of ASP on apoptosis proteins *in vivo*.** (**A**, **B**) Markers of apoptosis and the p38/MAPK signaling pathway were tested using Western blotting. (**C**, **D**) Markers of mitochondrial apoptosis and the p38/MAPK signaling pathway were tested using IHC (×400). The results for the treated group indicate that the medium-dose ASP treatment reduces mitochondrial apoptosis in BMNC cells. Note: ^*^P < 0.05 and ^**^P < 0.01 compared with the normal control group. ^▲^*P* < 0.05 and ^▲▲^*P* < 0.01 compared with the model control group.

The Bcl-2 expression of AA mice was significantly lower than NC group, whereas the expression of the proapoptotic protein Bax was prominently enhanced (Figure 7A, 7B). However, the abnormal levels of the Bax/Bcl-2 ratio, cleaved caspase-9 and cleaved caspase-3 were significantly recovered after treatment with 200 mg/kg/d ASP, not only in the WB test but also in the IHC test (Figure 7C, 7D).

### Preventing apoptosis in BMNCs by treatment with ASP

**Figure 8:**
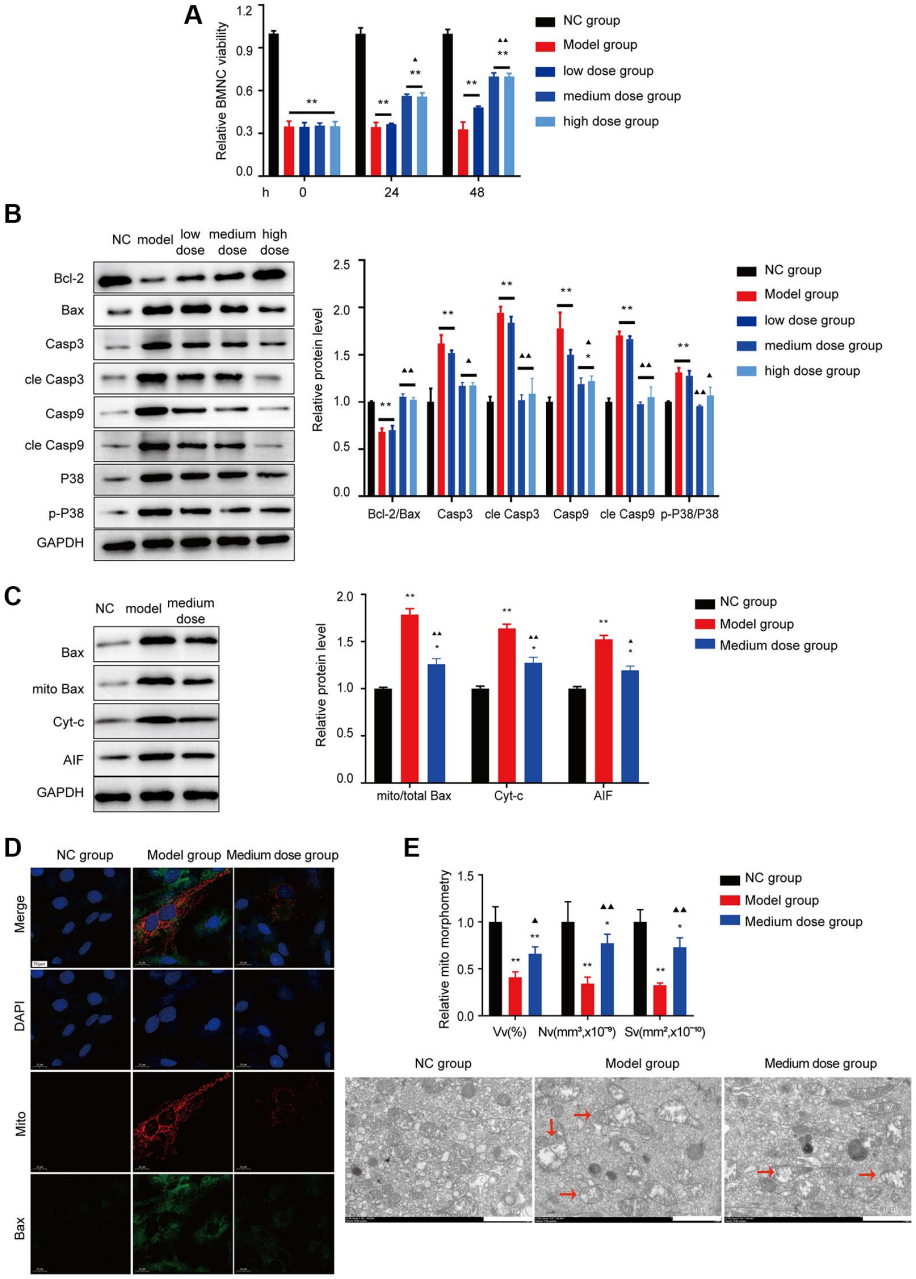
**Effects of ASP on apoptosis *in vitro*.** (**A**) BMNC cell viability was tested *in vitro*. (**B**) Markers of Bcl-2/Bax pathway apoptosis and the p38/MAPK signaling pathway were tested using Western blotting in BMNC cells. (**C**) Markers of Bax-associated mitochondrial apoptosis pathway were tested after treated with medium dose ASP. (**D**) The colocalization of Bax with mitochondria (yellow dots). (**E**) Mitochondrial structure was detected by electron microscopy. Note: ^*^P < 0.05 and ^**^P < 0.01 compared with the normal control group. ^▲^*P* < 0.05 and ^▲▲^*P* < 0.01 compared with the model control group.

As shown in Figure 8A, 8B, after treatment with 200 ng/ml ASP, BMNC cell counts were improved, and the expression levels of p38 associated apoptosis markers were significantly recovered (*P* < 0.01). The data demonstrated that ASP could decrease the levels of Cyt-C, Apaf-1, AIF and the Bax translocation level (Figure 8C). Then the mitochondrial colocalization with Bax was investigated. ASP alleviated the colocalization of Bax with mitochondria (Figure 8D). ASP also could improve the structure of mitochondria which damaged in model group (Figure 8E).

## DISCUSSION

The imbalance of the Th17/Treg ratio is associated with various immune-mediated diseases, including AA [19, 20]. Th17 immune response plays a role in the early phase of AA physiopathology [21], by secreting cytokines like IL-17, which is expanded in AA and associated with the severity of this disease [22, 23].

The evidence from benzene-induced AA mice [24] and X-ray exposure-induced AA mice [25] indicates that apoptosis and cell death-associated gene proportion of CD34+ bone marrow cells are very important mechanism in AA. The increased apoptosis in progenitor cells always associate with the immune system [26]. Study showed that tumour necrosis factor (TNF)-α and interleukin (IL)-17, which are secreted by Th17 cells, can induce apoptosis of haematopoietic stem/progenitor cells (HSPCs) [27, 28].

AS has been used for treating anaemia patients and chemotherapy like cyclophosphamide induced myelosuppression for many years in China [29]. AS is always combined with other herbs to compose the Danggui Buxue Tang to improve the hematopoietic function [30, 31], the mechanism includes attenuating apoptosis of HSC [30], regulating the differentiation of T lymphocytes [32], proliferating murine colony-forming unit-erthroid (CFU-E) and burst forming unit-erythroid (BFU-E) [33]. ASP can also exhibit anti-premature senescence of haematopoietic cells, via inhibiting oxidative stress [34] and secretion of IL-1, IL-6, TNF-α [35]. In addition to the anti-inflammatory activity of ASP [36], studies have shown the capacity of ASP to attenuate apoptosis via its antioxidant effects in various diseases [37, 38].

In this study, first, we demonstrated that AS can stimulate the bone marrow function recovery and alleviate the IL17A level (Figure 1), then higher levels of IL-17A and TGFBR1 were obtained in the GEO database (Figure 2A). IL-17 is secreted specifically by Th17 cells, a study verified that the concentration of IL-17 in the AA group was higher, whereas the concentration of TGF-β decreased [39]. TGFBR1 is a receptor TGF-β, which is why a higher level of TGFBR1 was detected in the GEO database considering that TGF-β is lower in AA. Then, the GSEA results revealed a close relationship between AA and inflammation and apoptosis (Figure 2D, 2E). Later, according to the active components of AS and analysed data using the network pharmacology method, we obtained core targets of AS in the treatment of AA. Analysis suggested that the genes were significantly enriched in the Th17 cell differentiation pathway, the core pathogenesis of AA (Figure 3).

For the mice model test, the AA mice showed statistically significant reductions in peripheral blood cells (Figures 4A and 6A) and BMNCs (Figure 4B), lower haematopoietic area (Figure 4D) and abnormal immune levels (Figure 4E–4G), which are clinical characteristics of AA. Our research obtained the same results as Lin’s and Shi’s study [39, 40]. The Treg/Th17 ratios and levels of IL-17A and TGF-β were abnormal. Under cellular stress by abnormal cytokines and ROS level (Figures 4G and 5A), activated p38 upregulates the expression of BH3-only proteins (such as Bad and BID). These BH3-only proteins can directly activate Bax, or inhibit anti-apoptotic proteins. Activated p38 can also phosphorylate and induce degradation of certain anti-apoptotic proteins. These stress-induced events result in the homo-oligomerization of the BCL2 effectors, leading to membrane permeabilization, release of cytochrome c, activation of caspase, increased Bax mitochondrial translocation, and ultimately cell death. AS can alleviated the apoptosis by abnormal cytokines like IL-17A (Figure 5C–5F).

After AS effect was confirmed (Figures 4, 5), we demonstrated the important component-ASP improved haematopoietic function (Figures 6–8). ASP is primarily composed of the monosaccharides glucose, mannose, galactose, rhamnose, arabinose, and xylose. It utilizes alpha (1, 4)-Glc Glycosidic linkage in its backbone and has functions such as regulating immunity [41, 42], anti-tumor effects [43], and protecting the gastrointestinal tract [44]. Although its specific composition and spatial conformation are not yet clear for studying its exact target, our work on the effectiveness of ASP can provide data support and research direction for pharmacological studies.

## CONCLUSION

**Figure 9:**
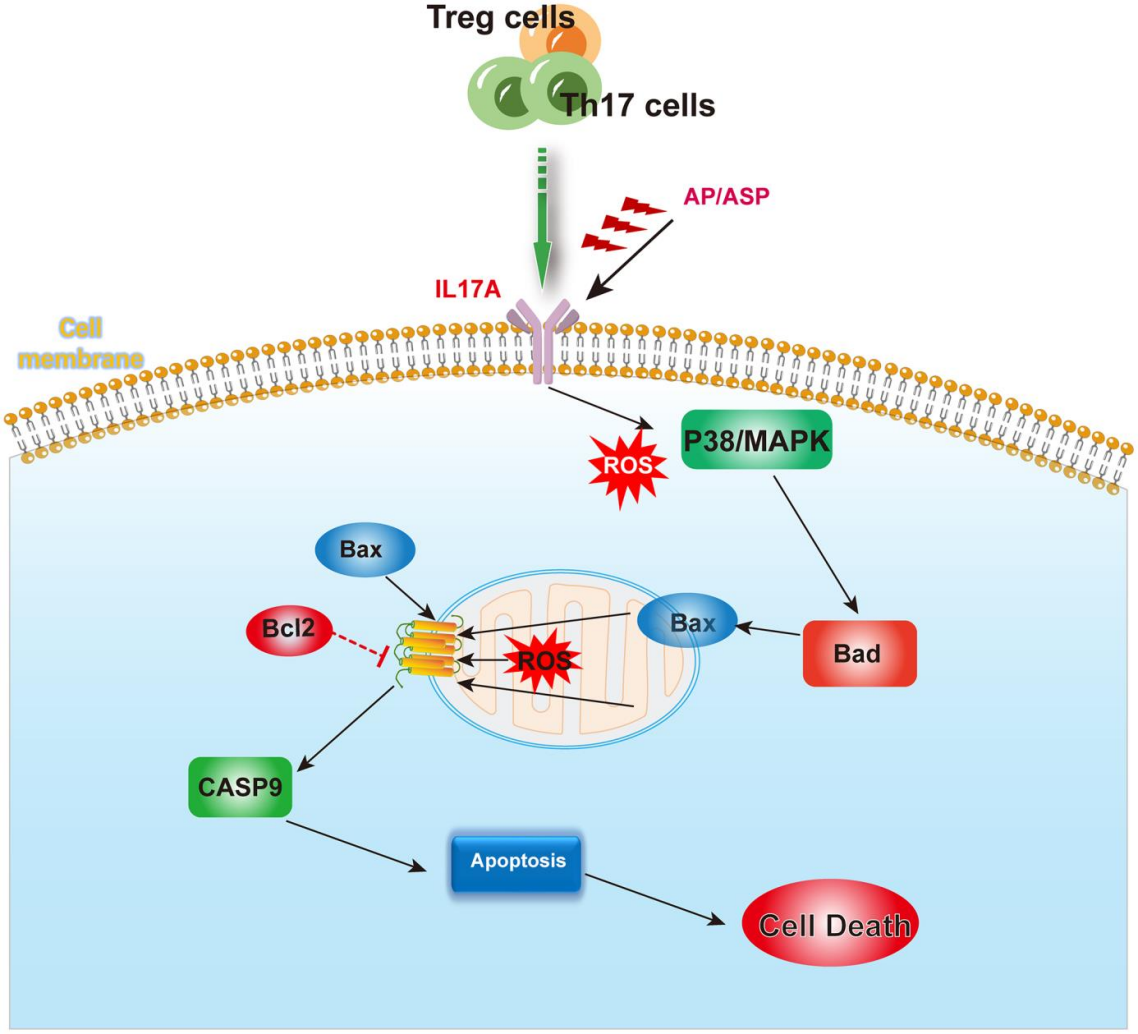
**The mechanism summary diagram.** AS and ASP could downregulate the expression of IL17A, and inhibit the activation of p38/MAPK pathway induced by abnormal cytokines and ROS, thereby ameliorating the aberrant levels of Bad, Bax and Bcl-2, and rectifying the abnormal apoptosis of bone marrow hematopoietic stem cells.

Based on clinical data, GEO dataset and mice experiment, we demonstrated that AS can reduce apoptosis to restore the function of hematopoietic stem cells by alleviating abnormal Treg/Th17 ratios and levels of IL-17A in AA. ASP has the same effect as AS (Figure 9).
